# Unravelling the role of APOE4 in vascular dysfunction and cognitive impairment

**DOI:** 10.1002/alz70855_099019

**Published:** 2025-12-23

**Authors:** Krystal K. Laing, Audrey Chagnot, Ross Lennen, Bethany Geary, William Mungall, Joanna M Wardlaw, Axel Montagne

**Affiliations:** ^1^ University of Edinburgh, Edinburgh, Scotland, United Kingdom; ^2^ UK Dementia Research Institute, University of Edinburgh, Edinburgh, Scotland, United Kingdom; ^3^ University of Dundee, Dundee, Scotland, United Kingdom; ^4^ Centre for Clinical Brain Sciences, The University of Edinburgh, Edinburgh, Scotland, United Kingdom

## Abstract

**Background:**

Apolipoprotein E (APOE) is a multifunctional lipid transporter, involved in lipoprotein metabolism and mediation of cholesterol homeostasis. The E4 variant of APOE is a primary genetic risk factor for Alzheimer's disease and has been associated with vascular conditions such as hyperlipidaemia, hypercholesterolemia, atherosclerosis and coronary artery disease (CAD). The mechanisms underlying APOE involvement in blood‐brain barrier breakdown (BBB‐B) are still poorly understood. In vivo, in vitro and clinical models of E4 have all demonstrated distinct profiling of molecular factors involved in ECM remodelling ‐ such as upregulation of PDGF‐BB in plasma and downregulation of VEGF in CSF. Investigating how APOE contributes to BBB‐B, through its function as a lipid transporter, is imperative to understanding the pathophysiology behind vascular contributions to dementia.

**Method:**

Longitudinal analyses characterizing the pathophysiology of humanized transgenic APOE knock‐in (KI) mice were performed utilizing behavioural analyses, immunohistochemistry, isolated microvessel and plasma proteomics, and magnetic resonance imaging (MRI). Cohorts of APOE3 and APOE4 (IHC, proteomics, and MRI with *n* =  4, 4, and 13 per timepoint and genotype, respectively) mice were assessed at multiple timepoints paralleling human age equivalencies indicative of early (2‐4 mo.), mature adult (7‐9 mo.), middle age (12‐14 mo.), and late life (17‐19 mo.).

**Result:**

Findings demonstrate genotype‐dependent and age‐related differences in vascular dynamics such as cerebral blood flow and BBB permeability. Protein quantification and immunohistochemical staining of APOE‐KI mice demonstrate dysregulation of pathways associated with mitochondrial functioning and cell proliferation, as well as differences in expression of key factors such as VEGFR2.

**Conclusion:**

This study provides additional support to previous reports that APOE4 contributes to impaired cerebrovascular function through mechanisms related to pericyte recruitment, vascular destabilization, and cognitive impairment. It may also provide additional insights for concepts which focus on staging of APOE‐related BBB breakdown.

